# Cost reduction associated with transradial access in percutaneous coronary intervention: A report from a Japanese nationwide registry

**DOI:** 10.1016/j.lanwpc.2022.100555

**Published:** 2022-08-12

**Authors:** Satoshi Shoji, Shun Kohsaka, Hiraku Kumamaru, Kyohei Yamaji, Shiori Nishimura, Hideki Ishii, Tetsuya Amano, Kiyohide Fushimi, Hiroaki Miyata, Yuji Ikari

**Affiliations:** a Department of Cardiology, Hino Municipal Hospital, Tokyo, Japan; b Department of Cardiology, Keio University School of Medicine, Tokyo, Japan; c Department of Healthcare Quality Assessment, The University of Tokyo Graduate School of Medicine, Tokyo, Japan; d Department of Cardiovascular Medicine, Kyoto University, Kyoto, Japan; e Department of Cardiovascular Medicine, Gunma University Graduate School of Medicine, Japan; f Department of Cardiology, Aichi Medical University, Aichi, Japan; g Department of Health Policy and Informatics, Graduate School of Medicine, Tokyo Medical and Dental University, Tokyo, Japan; h Department of Cardiology, Tokai University School of Medicine, Kanagawa, Japan

**Keywords:** Percutaneous coronary intervention, Transradial access, Cost, Nationwide registry

## Abstract

**Background:**

Percutaneous coronary intervention (PCI) is increasingly performed via transradial access (TRA). This study aimed to investigate the clinical and economic benefits of TRA compared with transfemoral access (TFA) under universal healthcare coverage system in Japan.

**Methods:**

A total of 36,153 patients (acute coronary syndrome [ACS], 15,266; stable ischemic heart disease [SIHD], 20,052) across 714 institutions in the Japanese nationwide PCI registry (J-PCI) in 2015 were analyzed (mean age 69.9 ± 11.1 years and 23.6% female). Cost was defined as the total amount of healthcare resources used to care for the patient during hospitalization. Propensity score matching analysis was conducted to balance the baseline characteristics of patients undergoing TRA and TFA.

**Findings:**

The median total cost of PCI was JPY 1,341,176 (interquartile range, 959,052), with higher expenses for ACS (JPY 1,772,116 [1,117,107]) compared with SIHD (JPY 1,119,153 [540,440]) patients. Most patients underwent PCI via TRA (73.8%), and after propensity score matching, TRA was associated with a reduced risk of in-hospital death and bleeding (0.88% vs. 1.91% [*P* < 0.0001] and 2.18% vs. 4.53% [*P* < 0.0001] in ACS, and 0.10% vs. 0.28% [*P* = 0.070] and 0.53% vs. 1.72% [*P* < 0.0001] in SIHD, respectively), which led to lower costs in both ACS (JPY 1,699,279 [1,164,554] for TRA vs. JPY 1,931,255 [1,070,222] for TFA; *P* < 0.0001), and SIHD (JPY 1,102,352 [505,904] for TRA vs. JPY 1,311,525 [706,450] for TFA; *P* < 0.0001) patients.

**Interpretation:**

In this direct cost analysis of a nationwide registry, the use of TRA was associated with cost saving for both ACS and SIHD patients.

**Funding:**

This study was funded by the Japan Society for the Promotion of Science (grant nos. 20H03915, 16H05215, 16KK0186, 20K22883, and 21K08064), Japan Agency for Medical Research and Development [AMED] (grant number 16lk1010004h0002), and the National Clinical Database. The J-PCI registry is led and supported by the Japanese Association of Cardiovascular Intervention and Therapeutics.

**Research in context**

**Evidence before this study**

Percutaneous coronary intervention is increasingly performed via transradial access (TRA). Previous analyses from the US, Europe, and developing countries suggested that TRA is associated with cost savings, mainly because of the lower complication rate and shorter length of stay compared with transfemoral access. However, these studies were conducted in an era or in areas where there is still a low implementation of TRA, with significant variations in its geographical uptake; hence, the learning curve of TRA procedures may have influenced the complication rate, length of stay, and costs.

**Added value of this study**

Nationwide analysis in Japan, where TRA is predominantly performed and its medical expense is covered under universal insurance system, demonstrated that TRA was associated with both cost savings and a reduced risk of complications.

**Implications of all the available evidence**

TRA may be a preferable choice for patients undergoing percutaneous coronary intervention. These findings provide policy makers with further scientific evidence to encourage the implementation of TRA, both from a clinical and from an economic perspective.

Alt-text: Unlabelled box

## Introduction

Percutaneous coronary intervention (PCI) remains widely employed in the management of coronary artery disease.^1^^,^^2^ The radial artery has become the standard vascular access site for the vast majority of PCIs.^3^ Compared with transfemoral access (TFA), transradial access (TRA) is associated with a reduced risk of major adverse events, including death, bleeding, acute kidney injury, and stroke in elective cases.^4^, ^5^, ^6^ TRA may also result in lower mortality in a subset of patients at increased risk, such as those presenting with acute coronary syndrome (ACS).^7^, ^8^, ^9^, ^10^ Accordingly, the current clinical guidelines of the United States (US), Europe, and Japan, all recommend the use of TRA over TFA regardless of patient risk.^11^^,^^12^

The adoption of TRA has dramatically increased globally since the publication of these studies and clinical guidelines (from 20% to over 70% in Japan and Europe).^3^^,^^13^ To further expedite the implementation of TRA, detailed analysis of the procedure-related healthcare cost is needed. Previous analyses from the US, Europe, and developing countries suggested that TRA is associated with cost savings mainly because of its lower complication rate and shorter length of stay (LOS) compared to TFA.^14^, ^15^, ^16^, ^17^, ^18^, ^19^ However, these studies may not be applicable to future economic predictions because they were conducted in areas with low TRA implementation and significant variations in geographical uptake; hence, the learning curve of TRA procedures may have influenced the complication rate, LOS, and costs.^20^ Furthermore, the economic analysis was conducted with a number of assumptions in the calculation of cost; for example, expenses related to device and pharmacotherapy had to be calculated from the average costs.^14^

We aimed to identify the economic benefits associated with the use of TRA in patients undergoing PCI by using the representative nationwide Japanese PCI registry (J-PCI). Our study is unique in that it was conducted using a nationwide registry in Japan, where TRA was introduced earlier than in Western countries and has become a standard of care.^21^^,^^22^ In addition, the Japanese healthcare system operates under universal healthcare coverage with a single-payer system, and the cost of care during hospitalization (including costs related to pharmacotherapy, laboratory and radiologic testing, nursing, invasive procedures and surgeries, anesthesia, blood transfusion, and rehabilitation, hospital rooms, and consultations) is recorded directly for individual patients, thus enabling the accurate calculation of cost using the same accounting system.^23^^,^^24^ Thus, understanding this economic analysis in Japan may help identify important cost-saving methods for other countries, and serves to lay a foundation for future quality improvement from the perspective of cost.

## Methods

### Data source

The J-PCI registry is an ongoing, prospective, multicenter, nationwide registry sponsored by the Japanese Association of Cardiovascular Intervention and Therapeutics (CVIT). The J-PCI has mandated consecutive registration for participating sites since 2013 and provides opportunities to study the representative Japanese PCI population.^2^^,^^25^ The registry collects information on patient background, clinical presentation, and angiographic and procedural details (the definitions of variables in the J-PCI are available online [http://www.cvit.jp/files/registry/j-pci-definition.pdf]).^26^ Cardiac catheterization procedures are performed in both publicly and privately funded hospitals in Japan, but because registration in the J-PCI registry is mandatory for the application of board certification and renewal under both systems, data completeness is over 90% (when compared with the annual reports from insurance claims data).^2^^,^^25^ Each hospital has a data manager who is responsible for collecting data and entering them into a computer database. The CVIT holds an annual meeting of data managers to secure appropriate data collection, and performs random audits (20 institutions annually) to check the quality of the abstracted data.

### Japanese healthcare system

The characteristics of the Japanese health insurance system include 1) mandatory coverage for anyone who permanently resides in Japan for three months or more (including both Japanese and non-Japanese citizens), and allows 2) enrollees to receive care from any medical provider regardless of plan. The current Japanese medical service fee system is operated under the Diagnosis Procedure Combination (DPC) payment system. The DPC system uses codes based primarily on combinations of diagnoses and procedures, similar to the diagnosis-related groups/prospective payment system (PPS) introduced in the US. As of April 2016, there were 4244 DPC codes. The Japanese system is unique in that it includes per diem, bundled/episode-based codes that are partially integrated with the fee-for-service (FSS) system. Medical fees for inpatient treatments that correspond to DPC diagnostic groups are calculated using a flat rate via the PPS, whereas fees for non-corresponding services are calculated using an FFS system. Basic hospitalizations, screenings (including diagnostic imaging), injections, medications, and procedures valued at less than JPY 10,000 are subject to bundled/episode-based evaluations. Surgeries, radiation therapy, anesthesia, and treatments valued at JPY 10,000 or more are not subject to bundled/episode-based evaluations. Fees for these treatments and services are calculated using the FFS system.

### Data linkage

The National Clinical Database (NCD), which serves as a platform for J-PCI and other procedure- or surgery-based databases, collects data from approximately 1100 participating hospitals. DPC includes information on all procedures, drugs, and medical materials used to treat the patients, as well as the associated cost.^27^^,^^28^ This social health insurance is accepted in all Japanese hospitals, thus enabling all participating hospitals to calculate costs by using the same accounting system. The prices of these procedures/equipment/materials, which are reflective of their costs, are unified on the basis of the national item-by-item FSS schedule by the Ministry of Health, Labour, and Welfare, with a strict ban on extra/discounted charges.^29^ We could estimate the cost of hospitalization care because all hospitals participating in this reimbursement system are required to produce this detailed per item record.^30^^,^^31^ Given that the DPC and NCD data do not have common personal identifiers, the data were linked using the patients’ sex, age, procedure type, and procedure date for each facility. Patients with more than one possible corresponding case were excluded from the linkage process.

The study protocol was approved by the Institutional Review Board Committee at the Network for the Promotion of Clinical Studies (a nonprofit organization affiliated with Osaka University Graduate School of Medicine, Osaka, Japan). Written informed consent was waived because of the retrospective study design. This study was approved by the Institutional Review Board of the University of Tokyo Graduate School of Medicine (review number 11467).

### Definition of important variables

Stable ischemic heart disease (SIHD)–related PCI was defined as scheduled PCIs that did not meet the criteria for urgent, emergency, or salvage PCI. PCI via the radial artery was defined as TRA, whereas PCI via the femoral artery was defined as TFA. PCI via the brachial artery was excluded from the study.

### Outcomes

The primary outcome was the total cost of hospitalization. The cost was defined as the entire amount of healthcare resources used to care for the patient, including all procedures, drugs, medical materials, and beds.^27^ The secondary outcome was in-hospital death and in-hospital bleeding requiring blood transfusion, which is defined as the transfusion of red cell concentrates (RCCs) conducted on the day after the procedure or later during hospitalization. In the DPC database, we could obtain information about the date of blood transfusion and procedures but not their timing; therefore, we could not distinguish the blood transfusion used before or after the procedure and whether blood transfusion was given on the same day as the procedure. Thus, we excluded same-day transfusion from the definition of in-hospital bleeding complications. We conducted a sensitivity analysis to assess the defined outcome, including the transfusion of RCCs on the same day.

### Statistical analyses

The demographics, in-hospital outcomes, and costs were compared between patients undergoing TRA versus TFA. Continuous variables with normal distributions were expressed as means ± standard deviation; other variables were expressed as medians [interquartile range]. Statistical comparisons of baseline characteristics and outcomes were performed using Student's *t*-test or the Mann–Whitney *U* test for continuous variables, and Pearson's chi-squared test or Fisher's exact test, as appropriate, for categorical variables.

First, we classified the patients into total cost quartiles and identified predictors for being in the highest quartile by using multivariable logistic regression analysis. The incorporated variables were age, sex, previous PCI, previous coronary artery bypass grafting, diabetes mellitus, current smoking, renal insufficiency, chronic lung disease, peripheral artery disease, previous heart failure, three-vessel disease, and left main trunk (LMT) lesion (all variables were extracted from the J-PCI registry).

Second, we conducted a propensity score (PS) matching analysis to assess the effect of TRA and TFA on costs and complications in a matched cohort (we performed this analysis among the ACS- and SIHD-related PCI populations individually, owing to the nature of different presentations), on the basis of the PSs calculated from all potentially unbalanced and clinically important covariates. The PS was calculated for each patient by using a logistic regression model that predicts selection into the TRA treatment group from variables that were considered important outcome risk factors and from factors used for selecting the access site: age, sex, previous PCI, previous coronary artery bypass grafting, previous myocardial infarction, diabetes mellitus, hypertension, dyslipidemia, current smoking, renal insufficiency, chronic lung disease, peripheral artery disease, previous heart failure, three-vessel disease, and LMT lesion (all variables were extracted from the J-PCI registry). Matching was performed with a 1:1 matching protocol without replacement, by using a caliper width equal to 0.01 of the standard deviation of the PS. The balance between the TRA and TFA groups in the matched cohort was estimated using absolute standardized difference. We compared the baseline characteristics and outcomes by using the PS-matched cohort. After PS matching, we used the paired t-test and McNemar's test to compare the paired samples. All statistical analyses were performed using SAS version 9.4 (SAS Institute, Cary, NC, USA).

### Role of the funding source

The funders played no role in the design and conduct of the study; collection, management, analysis, and interpretation of the data; review, or approval of the manuscript; and decision to submit the manuscript for publication.

## Results

### Study population

**Figure 1:**
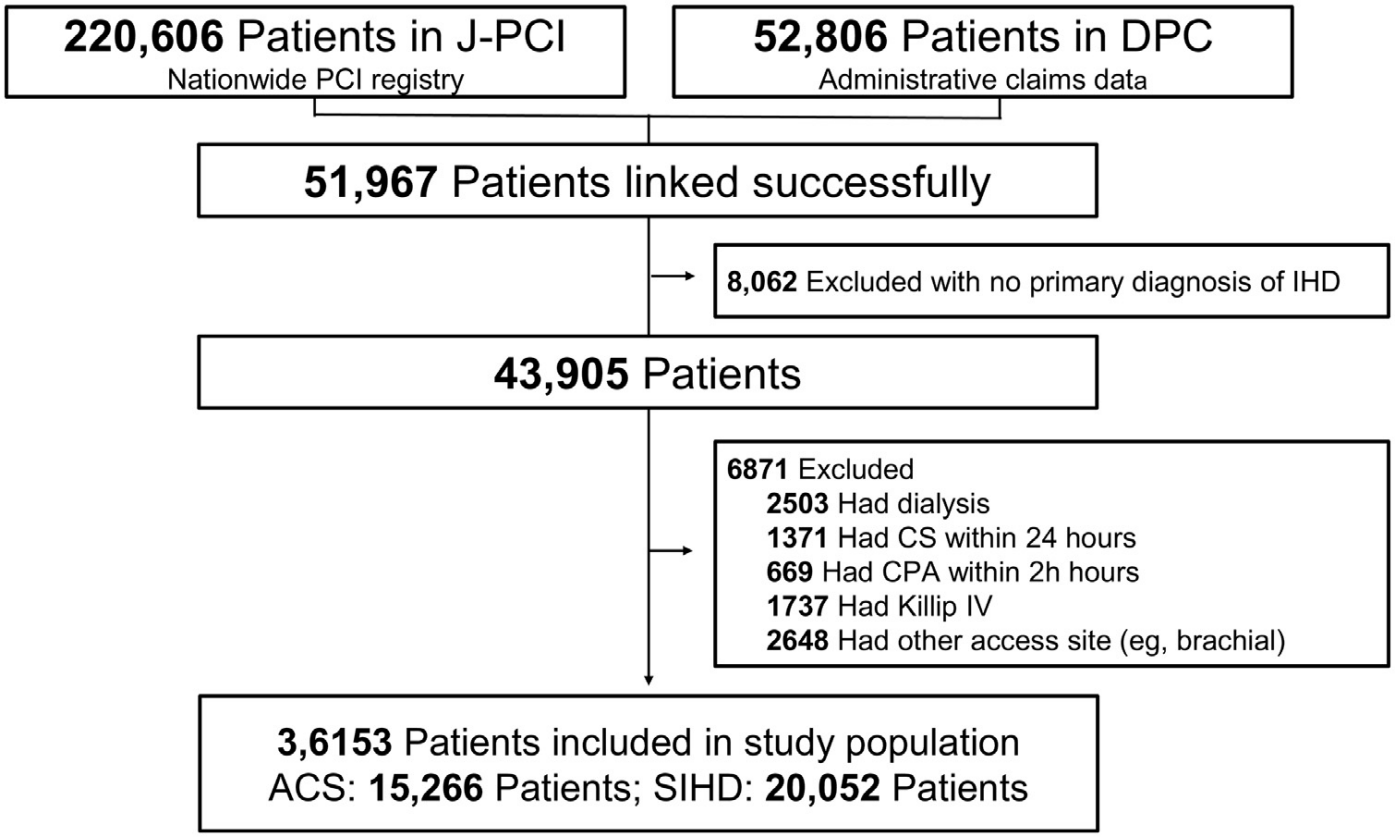
Study flowchart. ACS, acute coronary syndrome; CPA, cardiac pulmonary arrest; CS, cardiogenic shock; DPC, Diagnosis Procedure Combination; IHD, ischemic heart disease; J-PCI, Japanese percutaneous coronary intervention; SIHD, stable ischemic heart disease.

The patient-level data of 220,606 PCIs between January and December 2015 were extracted. We then linked the patient-level data of 52,806 PCIs from the DPC data available in NCD; 51,967 patients were linked successfully (Figure 1). Among the 51,967 patients, we excluded patients with no information on the primary diagnosis of ischemic heart disease (ICD codes: I20.0, I21.0, I21.1, I21.2, I21.3, I21.4, I21.9, I22.0, I22.1, I22.8, I22.9, I23.2, I23.3, I23.4, I23.5, I23.6, I24.0, I24.8, and I24.9) or SIHD (ICD codes: I20.1, I20.8. I20.9, I25.1, I25.5, I25.6, I25.8, and I25.9) in the DPC database (*n* = 8,062). All 43,905 consecutive PCI patients, including acute and elective cases, were initially included in this study. Figure 1 shows the exclusion criteria. We excluded patients undergoing hemodialysis (*n* = 2,503, 5.8%) and those who presented with cardiogenic shock (*n* = 1,371, 3.2%), out-of-hospital cardiac pulmonary arrest (*n* = 669, 1.5%), or Killip class IV (*n* = 1,737, 4.0%) because the application of TRA is not practical for these subgroups. Patients undergoing PCI via the brachial artery (*n* = 2,648, 6.0%) were also excluded. After exclusion, the final study population consisted of 36,153 PCI patients (ACS, 15,266; SIHD, 20,052).

Among the 36,153 patients (mean age of 69.9 ± 11.1 years; 23.6% were female), 26,695 (73.8%) underwent TRA, whereas 9,458 (26.2%) underwent TFA (Table 1). No significant difference in age was observed between patients with TRA and TFA (69.9 ± 11.0 vs. 70.0 ± 11.4 years, *P* = 0.366). Patients with TRA were more likely to be male and were less likely to have chronic kidney disease, diabetes mellitus, and previous heart failure. In addition, patients who underwent TRA were less likely to experience in-hospital death and in-hospital bleeding than patients who underwent TFA (0.44% vs. 1.29% for in-hospital death [*P* < 0.0001], and 0.99% vs. 3.32% for in-hospital bleeding [*P* < 0.0001]).

**Table 1:** Baseline characteristics, outcomes, and total cost of patients undergoing TRA and TFA.

	Total	TFA	TRA
	*n*=36,153	*n*=9,458	*n*=26,695
Age (years)	69.9 ±11.1	70.0 ±11.4	69.9 ±11.0
Male	27,626 (76.4)	7064 (74.7)	20,562 (77.0)
Previous PCI	15,868 (43.9)	3826 (40.5)	12,042 (45.1)
Previous CABG	1249 (3.5)	512 (5.4)	737 (2.8)
Previous MI	7518 (20.8)	2051 (21.7)	5467 (20.5)
Diabetes mellitus	14,977 (43.4)	4003 (44.4)	10,974 (43.1)
Hypertension	26,895 (78.0)	6990 (77.6)	19,905 (78.1)
Dyslipidemia	23,939 (69.4)	6150 (68.3)	17,789 (69.8)
Current smoker	12,037 (34.9)	3296 (36.6)	8741 (34.3)
Renal insufficiency	4222 (12.2)	1255 (13.9)	2967 (11.7)
Chronic lung disease	790 (2.3)	213 (2.4)	577 (2.3)
Peripheral artery disease	2055 (6.0)	546 (6.1)	1509 (5.9)
Previous heart failure	3176 (8.8)	937 (9.9)	2239 (8.4)
Three vessel disease	4299 (11.9)	1320 (14.0)	2979 (11.2)
LMT	1283 (3.5)	521 (5.5)	762 (2.9)
ACS	15,266 (42.2)	4986 (52.7)	10,280 (38.5)
STEMI	7540 (20.9)	3100 (32.8)	4440 (16.6)
NSTE-ACS	7726 (21.4)	1886 (19.9)	5840 (21.9)
Length of stay	4 [9]	7 [11]	3 [7]
All complication	716 (1.98)	306 (3.24)	410 (1.54)
In-hospital death	240 (0.66)	122 (1.29)	118 (0.44)
In-hospital bleeding (main outcome: transfusion after 1POD)	579 (1.60)	314 (3.32)	265 (0.99)
In-hospital bleeding (sub-outcome: including the same-day transfusion)	769 (2.13)	418 (4.42)	351 (1.31)
Total cost	1,341,176 [959,052]	1,645,218 [1,052,029]	1,244,119 [849,859]

Values are expressed as mean ± standard difference or median [interquartile range] or *n* (%). Abbreviations: CABG, coronary artery bypass grafting; LMT, left main trunk; MI, myocardial infarction; NSTE-ACS, non-ST-elevation acute coronary syndrome; PCI, percutaneous coronary intervention; POD, postoperative day; STEMI, ST-elevated myocardial infarction; TFA, transfemoral access; TRA, transradial access.

### Cost distribution

**Figure 2:**
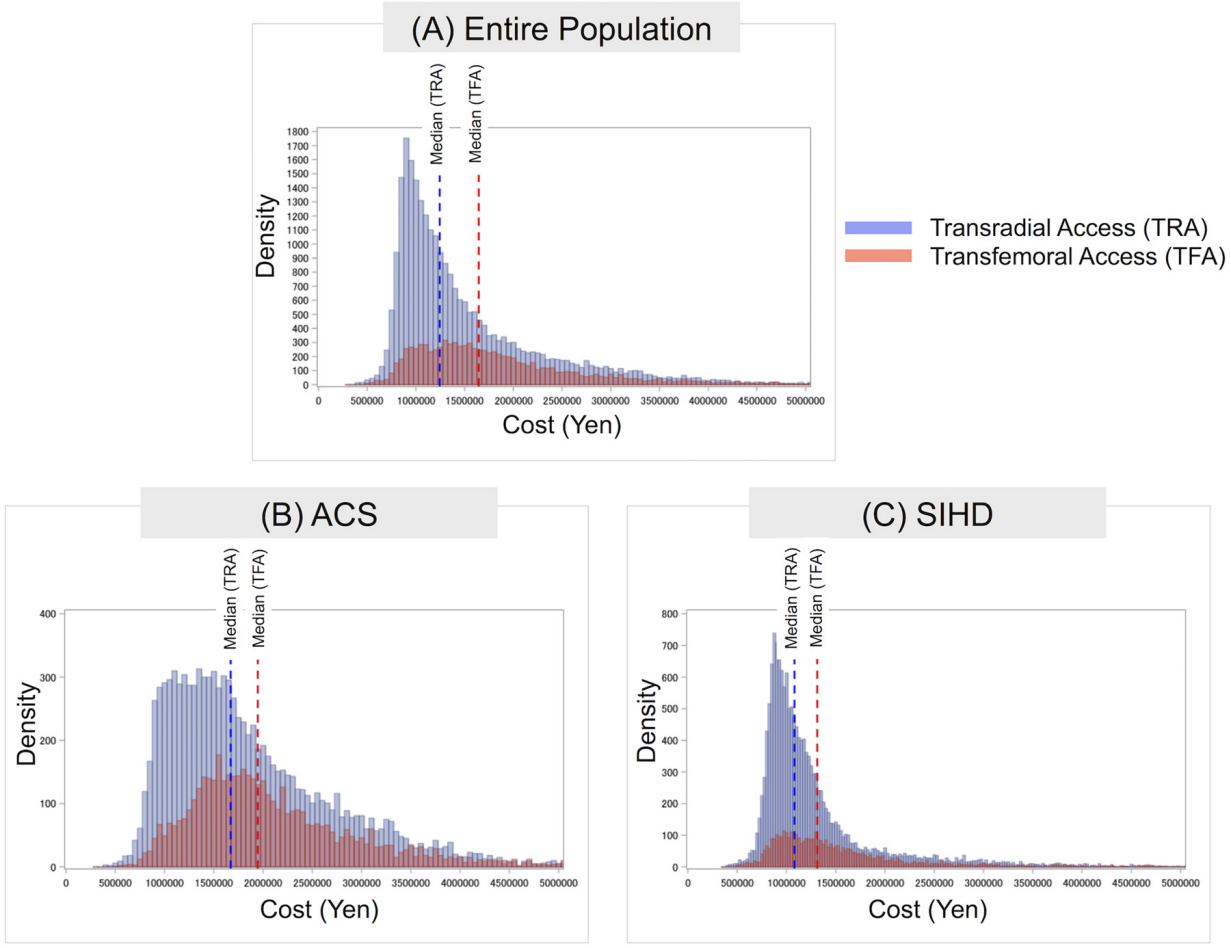
Cost distribution for transradial access and transfemoral access in the (A) entire population, (B) patients with acute coronary syndrome (ACS), and (C) patients with stable ischemic heart disease (SIHD).

The cost distribution among the entire population and the cost distribution stratified by the access site among the entire population are shown in Figure 2A. The median total cost of PCI was JPY 1,341,176 [959,052]. Patients with TRA had lower costs than patients undergoing TFA (JPY 1,244,119 [849,859] for TRA vs. JPY 1,645,218 [1,052,029] for TFA; *P* < 0.0001 [Figure 2A, Table 1]). Cost distribution among patients with ACS and with SIHD are shown in Figure 2B and C. Overall, the median cost of TFA was significantly higher and had substantial variations.

### Regression analysis

Among the 15,266 patients with ACS, three-vessel disease (OR, 2.77; 95% CI, 2.51–3.07), LMT lesion (OR, 2.21; 95% CI, 1.82–2.68), previous heart failure (OR, 1.57; 95% CI, 1.34–1.83), previous PCI (OR, 1.51; 95% CI, 1.38–1.65), renal insufficiency (OR, 1.34; 95% CI, 1.19–1.50), current smoking (OR, 1.14; 95% CI, 1.04–1.24), and age (OR, 1.006; 95% CI, 1.003–1.010), were independent predictors of the highest cost quartile (Supplemental Figure 1). Conversely, previous coronary artery bypass grafting (OR, 0.50; 95% CI, 0.37–0.67), TRA (OR, 0.74; 95% CI, 0.68–0.80), and male (OR, 0.88; 95% CI, 0.80–0.97), were independent predictors of a decrease in highest cost quartile (Supplemental Figure 1). Similarly, among the 20,052 patients with SIHD, three-vessel disease (OR, 2.10; 95% CI, 1.90–2.32), LMT lesion (OR, 2.09; 95% CI, 1.79–2.44), previous heart failure (OR, 1.33; 95% CI, 1.20–1.47), current smoking (OR, 1.32; 95% CI, 1.22–1.42), peripheral artery disease (OR, 1.19; 95% CI, 1.05–1.34), renal insufficiency (OR, 1.14; 95% CI, 1.04–1.26), and age (OR, 1.007; 95% CI, 1.004–1.011), were independent predictors of the highest quartile (Supplemental Figure 2). Conversely, TRA (OR, 0.45; 95% CI, 0.42–0.48) and male (OR, 0.85; 95% CI, 0.78–0.92), were independent predictors of a decrease in highest cost quartile (Supplemental Figure 2).

### PS analysis

The first PS matching generated 4,546 pairs of patients among the ACS-related PCI population. After PS matching, there were minimal differences between the two groups, with a standardized mean difference of less than 0.2 for all variables included in the PS model (Table 2, Supplemental Figure 3). In the PS-matched cohort, TRA was associated with a reduced risk of in-hospital death (0.88% [TRA] vs. 1.91% [TFA]; *P* < 0.0001) and in-hospital bleeding (2.18% [TRA] vs. 4.53% [TFA] for the main outcome [transfusion after one postoperative day], and 2.68% [TRA] vs. 5.74% [TFA] for the sub-outcome [including the same-day transfusion]; *P* < 0.0001; Table 3). Furthermore, TRA was also associated with lower costs than TFA (JPY 1,699,279 [1,164,554] for TRA vs. JPY 1,931,255 [1,070,222] for TFA; *P* < 0.0001) among the ACS population (Table 3).

**Table 2:** Baseline characteristics of patients with acute coronary syndrome undergoing TRA and TFA before and after propensity score matching.

	Before matching			After matching		
	TFA	TRA	Standardized difference	TFA	TRA	Standardized difference
	*n* = 4,986	*n* = 10,280		*n* = 4,546	*n* = 4,546	
Age (years)	69.3 ±12.3	68.7 ±12.4	−0.040	68.9 ±12.3	68.9 ±12.9	−0.004
Male	3685 (73.9)	7925 (77.1)	−0.035	3397 (74.7)	3428 (75.4)	−0.058
Previous PCI	1115 (22.4)	2772 (27.0)	−0.114	1021 (22.5)	1151 (25.3)	−0.066
Previous CABG	145 (2.91)	137 (1.33)	0.107	78 (1.7)	71 (1.6)	0.011
Previous MI	735 (14.7)	1565 (15.2)	−0.019	644 (14.2)	769 (16.9)	−0.077
Diabetes Mellitus	1791 (38.2)	3674 (38.0)	0.003	1726 (38.0)	1956 (43.0)	−0.104
Hypertension	3479 (74.2)	7238 (74.8)	−0.015	3369 (74.1)	3212 (70.7)	0.079
Dyslipidemia	3024 (64.5)	6392 (66.1)	−0.034	2927 (64.4)	22,971 (65.3)	−0.020
Current smoker	1929 (41.1)	3956 (40.9)	0.002	1880 (41.4)	1833 (40.3)	0.021
Renal insufficiency	617 (13.2)	1013 (10.5)	0.081	563 (12.4)	619 (13.6)	−0.038
Chronic lung disease	119 (2.5)	200 (2.1)	0.031	111 (2.4)	131 (2.9)	−0.029
Peripheral artery disease	181 (3.9)	334 (3.5)	0.021	169 (3.7)	196 (4.3)	−0.032
Previous heart failure	315 (6.3)	536 (5.2)	0.049	263 (5.8)	312 (6.9)	−0.046
Three vessel disease	721(14.5)	1396 (13.6)	0.017	631 (13.9)	824 (18.1)	−0.122
LMT	216 (4.3)	287 (2.8)	0.079	162 (3.6)	184 (4.1)	−0.026

Values are expressed as mean ± standard difference or median [interquartile range] or *n* (%). Abbreviations: CABG, coronary artery bypass grafting; LMT, left main trunk; MI, myocardial infarction; PCI, percutaneous coronary intervention; TFA, transfemoral access; TRA, transradial access.

**Table 3:** Ischemic and bleeding outcomes and total cost of patients with acute coronary syndrome undergoing TRA and TFA before and after propensity score matching.

	Before matching			After matching		
	TFA	TRA	*P* value	TFA	TRA	*P* value
	*n* = 4,986	*n* = 10,280		*n* = 4,546	*n* = 4,546	
In-hospital death	105 (2.11)	100 (0.97)	<0.0001	87 (1.91)	40 (0.88)	<0.0001
In-hospital bleeding (main outcome: transfusion after 1POD)	231 (4.63)	189 (1.84)	<0.0001	206 (4.53)	99 (2.18)	<0.0001
In-hospital bleeding (sub-outcome: including the same-day transfusion)	293 (5.88)	244 (2.37)	<0.0001	261 (5.74)	122 (2.68)	<0.0001
Total cost	1,947,542 [1,092,196]	1,671,817 [1,120,685]	<0.0001	1,931,255 [1,070,222]	1,699,279 [1,164,554]	<0.0001

Values are expressed as *n* (%) or median [interquartile range]. Abbreviations: POD, postoperative day; TFA, transfemoral access; TRA, transradial access.

The second PS matching generated 3,957 pairs among patients with SIHD (Table 4, Supplemental Figure 4). Directionally similar to the first PS-matching analysis, TRA was associated with a reduced risk of in-hospital death (0.10% [TRA] vs. 0.28% [TFA]; *P* = 0.070) and in-hospital bleeding (0.53% [TRA] vs. 1.72% [TFA]; *P* < 0.0001) (Table 5). TRA was also associated with lower costs than TFA (JPY 1,102,352 [505,904] for TRA vs. JPY 1,311,525 [706,450] for TFA; *P* < 0.0001) among patients with SIHD (Table 5).

**Table 4:** Baseline characteristics of patients with stable ischemic heart disease undergoing TRA and TFA before and after propensity score matching.

	Before matching			After matching		
	TFA	TRA	Standardized difference	TFA	TRA	Standardized difference
	*n* = 4,258	*n* = 15,794		*n* = 3,957	*n* = 3,957	
Age (years)	70.8 ± 10.3	70.6 ± 10.0	−0.016	70.7 ± 10.2	70.4 ± 11.0	−0.035
Male	3218 (75.6)	12,274 (76.8)	−0.035	2991 (75.6)	3089 (78.1)	−0.058
Previous PCI	2625 (61.7)	9033 (56.6)	0.103	2457 (62.1)	2195 (55.5)	0.135
Previous CABG	359 (8.4)	587 (3.7)	0.205	291 (7.4)	297 (7.5)	−0.006
Previous MI	1280 (30.1)	3791 (23.7)	0.147	1191 (30.1)	1144 (28.9)	0.026
Diabetes Mellitus	2121 (51.5)	7113 (46.3)	0.104	2025 (51.2)	2079 (52.5)	−0.027
Hypertension	3346 (81.3)	12,326 (80.2)	0.029	3227 (81.6)	2973 (75.1)	0.163
Dyslipidemia	3000 (72.9)	11,079 (72.1)	0.020	2895 (73.2)	2735 (69.1)	0.091
Current smoker	1282 (31.2)	4636 (30.2)	0.024	1236 (31.2)	1229 (31.1)	0.003
Renal insufficiency	609 (14.8)	1910 (12.4)	0.065	578 (14.6)	733 (18.5)	−0.114
Chronic lung disease	92 (2.2)	367 (2.4)	−0.009	90 (2.3)	145 (3.7)	−0.009
Peripheral artery disease	349 (8.5)	1157 (7.5)	0.037	336 (8.5)	476 (12.0)	−0.130
Previous heart failure	590 (13.9)	1663 (10.4)	0.110	555 (14.0)	640 (16.2)	−0.065
Three vessel disease	576 (13.5)	1532 (9.6)	0.117	516 (13.0)	562 (14.2)	−0.036
LMT	293 (6.9)	466 (2.9)	0.187	229 (5.8)	225 (5.7)	0.005

Values are expressed as mean ± standard difference or median [interquartile range] or *n* (%). Abbreviations: CABG, coronary artery bypass grafting; LMT, left main trunk; MI, myocardial infarction; PCI, percutaneous coronary intervention; TFA, transfemoral access; TRA, transradial access.

**Table 5:** Ischemic and bleeding outcomes and total cost of patients with stable ischemic heart disease undergoing TRA and TFA before and after propensity score matching.

	Before matching			After matching		
	TFA	TRA	*P* value	TFA	TRA	*P* value
	*n* = 4,258	*n* = 15,794		*n* = 3,957	*n* = 3,957	
In-hospital death	11 (0.26)	15 (0.09)	0.008	11 (0.28)	4 (0.10)	0.070
In-hospital bleeding (main outcome: transfusion after 1POD)	74 (1.74)	68 (0.43)	<0.0001	68 (1.72)	21 (0.53)	<0.0001
In-hospital bleeding (sub-outcome: including the same-day transfusion)	113 (2.65)	95 (0.59)	<0.0001	105 (2.65)	26 (0.66)	<0.0001
Total cost	1,311,828 [716,027]	1,080,722 [467,050]	<0.0001	1,311,525 [706,450]	1,102,352 [505,904]	<0.0001

Values are expressed as *n* (%) or median [interquartile range]. Abbreviations: POD, postoperative day; TFA, transfemoral access; TRI, transradial access.

## Discussion

By using a nationwide registry in Japan, where TRA is predominantly performed and its medical expense is covered under a universal insurance system, we identified that the median cost of TFA was significantly higher than that of TRA with substantial variations. After PS-matching, the use of TRA was associated with cost savings for both ACS and SIHD patients. After rigorous statistical adjustments we also demonstrated that TRA was associated with a reduced risk of complications. Thus, our study suggests that TRA may be a preferable choice for patients undergoing PCI, both from an economic and from a clinical perspective.

Our study is within the context of other studies that have evaluated the economic benefits of TRA. Amin et al.^16^ reported similar results (*n* = 7121), with total cost savings of USD 830 (95% CI, USD 296–1364; *P* < 0.001) for TRA versus TFA that increased as the risk of bleeding increased, with cost savings calculated at low risk (USD 642 [95% CI, USD 43–1236; *P* = 0.035]); moderate risk: USD 706 (95% CI, USD 104–1308; *P* = 0.029); and high risk: USD 1621 (95% CI, USD 271–2971; *P* = 0.039). Amin et al.^32^ also conducted an analysis on 434,172 low-risk, uncomplicated ACS patients eligible for early discharge. In low-risk, uncomplicated patients with ST-elevated myocardial infarction, compared with TFA and hospital stay ≥ 3 days, a TRA strategy with hospital stay < 3 days and TFA with hospital stay < 3 days were associated with cost savings of USD 6206 and USD 4802, respectively. From a non-US perspective, Jin et al.^18^ reported a lower total adjusted cost for TRA compared with TFA (adjusted difference USD 1283) in a study of 5306 patients in China. Mamas et al.^14^ conducted a nationwide large-scale analysis (*n* = 323,655) and found that TRA offered an average cost saving of GBP 250.59 per procedure (22% reduction) compared with TFA, with the majority of cost savings derived from a reduced LOS (GBP 190.43) rather than direct costs of complications (GBP 3.71). If operators had adopted TRA at the rate of the region with the highest utilization, cost savings of GBP 33.40 million could have been achieved in the United Kingdom. However, these studies may not be applicable to future economic predictions because they were conducted in areas where there is still a low implementation of TRA with significant variations.^20^ Therefore, the learning curve of TRA procedures may have influenced the complication rate, LOS, and costs. Our study is unique in that it was conducted using a nationwide registry in Japan, where TRA was introduced earlier than in Western countries and has become a standard of care, mainly because of the preference of interventionists and patients for the non-invasive nature of TRA.^21^^,^^22^ If all interventionists adopted TRA instead of TFA (a total of 63,782 cases were conducted using TFA in Japan in 2015), approximately JPY 13 billion in cost savings could have been achieved. Given that the implementation of TRA is set to increase in other countries (US, Europe, Australia,^17^ and developing countries) in the next decade, this economic analysis in Japan may help identify important cost-saving methods for other countries, and serves to lay a foundation for future quality improvements from a cost perspective.

Interestingly, our current analysis found that the greatest cost saving was obtained in patients with SIHD (12% less costly in ACS and 17% less costly in SIHD), and this result is inconsistent with a previous study that showed that the greatest cost saving was obtained for high risk patients presented with ACS.^14^ The detailed reasons underlying this discrepancy are unclear but may be driven by the use of a vascular closure device (we observed that a substantial number of TFA cases used a vascular closure device), which costs JPY 3000 more than a manual or radial compression device. Nonetheless, this finding is acceptable for most interventionists who are not familiar with TRA because they could start the radial approach from relatively low-risk situations. Further efforts to shorten the LOS are required (e.g., same-day discharge) to save costs in Japan, where an aging society necessitates the appropriate use of healthcare resources and expenditures.

Despite the proven advantages of TRA, the use of TRA in developing countries has remained relatively low with substantial variations in its geographical uptake.^18^^,^^19^^,^^33^, ^34^, ^35^, ^36^ The associate concerns include the increased risk of complications and longer procedural times with less experienced interventionists, as well as the learning curve for TRA. Furthermore, poor awareness for quality metrics (e.g., TRA, door-to-balloon time ≤90 min for ST-elevation myocardial infarction, and guideline-directed medical therapy, which are all recommended in clinical practice guidelines) could be another reason associated with the low adoption of TRA in developing countries.^34^ Understanding these barriers (less experience to TRA and low awareness to quality metrics) that have so far obstructed the rapid adoption of TRA in developing countries may help facilitate its adoption in these countries. Our study contributes to the growing body of literature that provides developing countries with opportunities to nurture and accelerate the utilization of TRA.

### Limitations

Several limitations should be considered when interpreting the results of this study. First, considering that this is an observational study, there may be unknown and unmeasurable factors that confound the relationship between access site and postprocedural complications and cost (e.g., Charlson comorbidity index). Second, costs in our analysis are not the actual amounts billed to the payer or the patients, because DPC is a per diem bundled payment system based on diagnosis for hospitalization. Nevertheless, the recorded costs represent what would have been billed if the hospital was not enrolled in the bundled payment system, and DPC includes information on all procedures, drugs, and medical materials used to treat the patients, as well as the associated cost. Furthermore, Japan has universal health insurance coverage, and virtually all Japanese individuals are currently covered by social health insurance. This social health insurance is accepted in all Japanese hospitals, thus enabling all participating hospitals to calculate costs by using the same accounting system. Third, we did not capture the number of crossover cases switching from TRA to TFA. However, our previous study reported that access site crossover cases from TRA to TFA were observed only in 2.9% of patients undergoing TRA.^37^ In this study, the anatomical variation of the radial artery was an independent predictor of transradial procedure failure, and most crossover cases changed the access site before the catheters or wires could reach the aorta. Therefore, crossover cases required few additional costs, and it is acceptable for the analysis that most crossover cases were included in the TFA group. Fourth, we were not able to include costs in the outpatient setting, such as preprocedural stress testing or coronary computed tomography angiography. However, given that the selection of the access site would not be biased by the preprocedural testing, it would not affect the association between the access site and in-hospital cost. Fifth, the selection of the access site should be discussed using a “heart–kidney” team discussion for patients with advanced chronic kidney disease, because TRA is associated with a small increase in the risk of radial artery occlusion.^38^ Sixth, while all data of patients included in the J-PCI registry were available (*N*=220,606), NCD data (administrative coding and cost data) were available from the hospitals that voluntarily participated in the NCD linkage program (*N*=52,806). Finally, not all institutions in Japan participate in the J-PCI registry. Nonetheless, because the J-PCI registry covers over 90% of all patients undergoing PCI,^2^ we believe that the effect of expertise on radial technique or local preference, such as the use of a vascular closure device, could be minimized.

### Conclusions

By using a nationwide registry in Japan, where TRA is predominantly performed, we demonstrated that TRA was associated with cost savings and a reduced risk of complications for both ACS and SIHD cases. TRA may therefore be a preferable choice for patients undergoing PCI. These findings provide policy makers with further scientific evidence to encourage the implementation of TRA, both from a clinical and from an economic perspective.

## Contributors

Dr. Kohsaka takes responsibility for the integrity of the data and the accuracy of the data analysis.

Concept and design: Shoji, Kohsaka, Ikari.

Acquisition, analysis, or interpretation of data: Shoji, Kohsaka, Kumamaru, Nishimura, Ikari.

Drafting of the manuscript: Shoji, Kohsaka, Ikari.

Critical revision of the manuscript for important intellectual content: Ymaji, Ishii, Amano.

Statistical analysis: Shoji, Kumamaru, Nishimura.

Obtained funding: Shoji, Kohsaka.

Administrative, technical, or material support: Amano, Fushimi, Miyata, Ikari.

Supervision: Amano, Fushimi, Miyata, Ikari.

## Data sharing statement

The data and materials used to conduct this research are available to researchers for purposes of reproducing the results or replicating the procedure on request. The procedure does need to follow the Act on the Protection of Personal Information Law (as of May 2017) and the Ethical Guidelines for Medical and Health Research Involving Human Subjects (as of March 2015) in Japan.

## Declaration of interests

SK received investigator-initiated grant funding from Novartis, Daiichi-Sankyo, and Bristol-Myers Squibb and personal fees from Bristol-Myers Squibb and Pfizer. HK, SN and HM are affiliated with the Department of Healthcare Quality Assessment. The department is a social collaboration department supported by National Clinical Database, Johnson & Johnson KK, and Nipro corporation. HK has received consultation fee from Mitsubishi Tanabe Pharma, and speaker fee from Johnson & Johnson KK. H.I. receives lecture fees from Astellas, AstraZeneca, Bayer, Bristol-Myers Squibb, Daiichi Sankyo, Kowa, Mochida, Novartis, Otsuka, Pfizer, and Tanabe-Mitsubishi. All other authors have no relationships relevant to the contents of this paper.

## Appendix Supplementary materials

Image, application 1
