# Supplementary material for: Cost reduction associated with transradial access in percutaneous coronary intervention: A report from a Japanese nationwide registry
Source: Lancet Reg Health West Pac. 2022 Aug 12;28:100555. doi: 10.1016/j.lanwpc.2022.100555 (PMC9391571; doi:10.1016/j.lanwpc.2022.100555)
Supplement: Supplementary file 1 [file mmc1.docx]

**Supplemental Materials**

**Supplemental Figure 1.** Independent predictors associated with the highest cost quartile among patients with acute coronary syndrome.


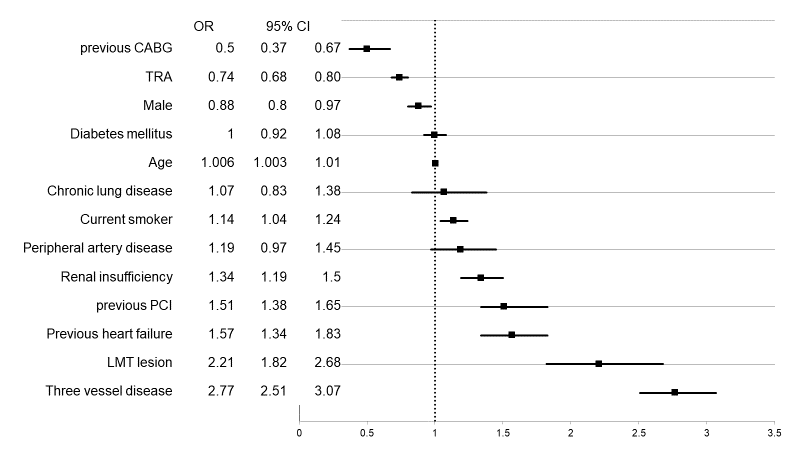


CABG, coronary artery bypass grafting; CI, confidence interval LMT, left main trunk; OR, odds ratio; PCI, percutaneous coronary intervention; TRA, transradial access.

**Supplemental Figure 2.** Independent predictors associated with the highest cost quartile among patients with elective percutaneous coronary intervention (PCI).


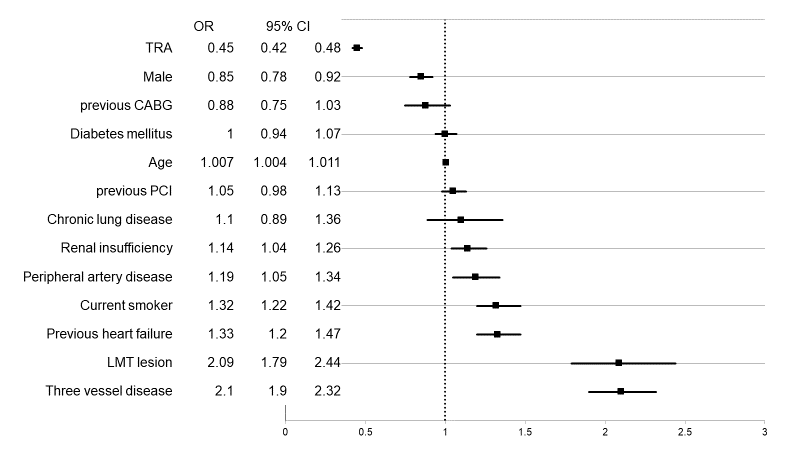


CABG, coronary artery bypass grafting; CI, confidence interval LMT, left main trunk; OR, odds ratio; TRA, transradial access.

**Supplemental Figure 3.** Standardized mean difference before and after matching among patients with acute coronary syndrome.


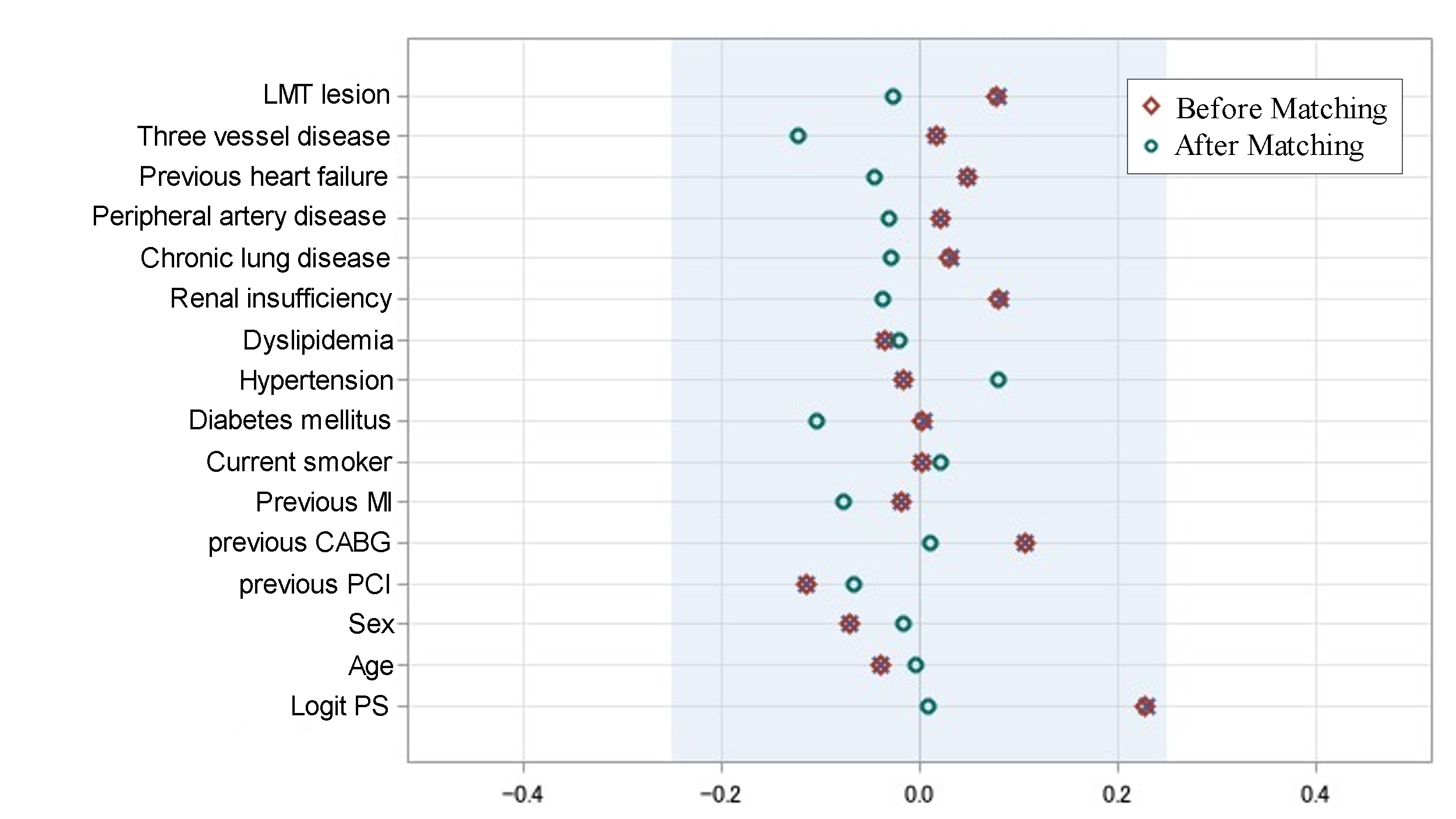


**Variables Included in the Propensity Score (PS):** age, sex, previous percutaneous coronary intervention (PCI), previous coronary artery bypass grafting (CABG), previous myocardial infarction (MI), diabetes mellitus, hypertension, dyslipidemia, current smoking, renal insufficiency, chronic lung disease, peripheral artery disease, previous heart failure, three-vessel disease, and left main trunk (LMT) lesion.

**Supplemental Figure 4.** Standardized mean difference before and after matching among patients with stable ischemic heart disease.


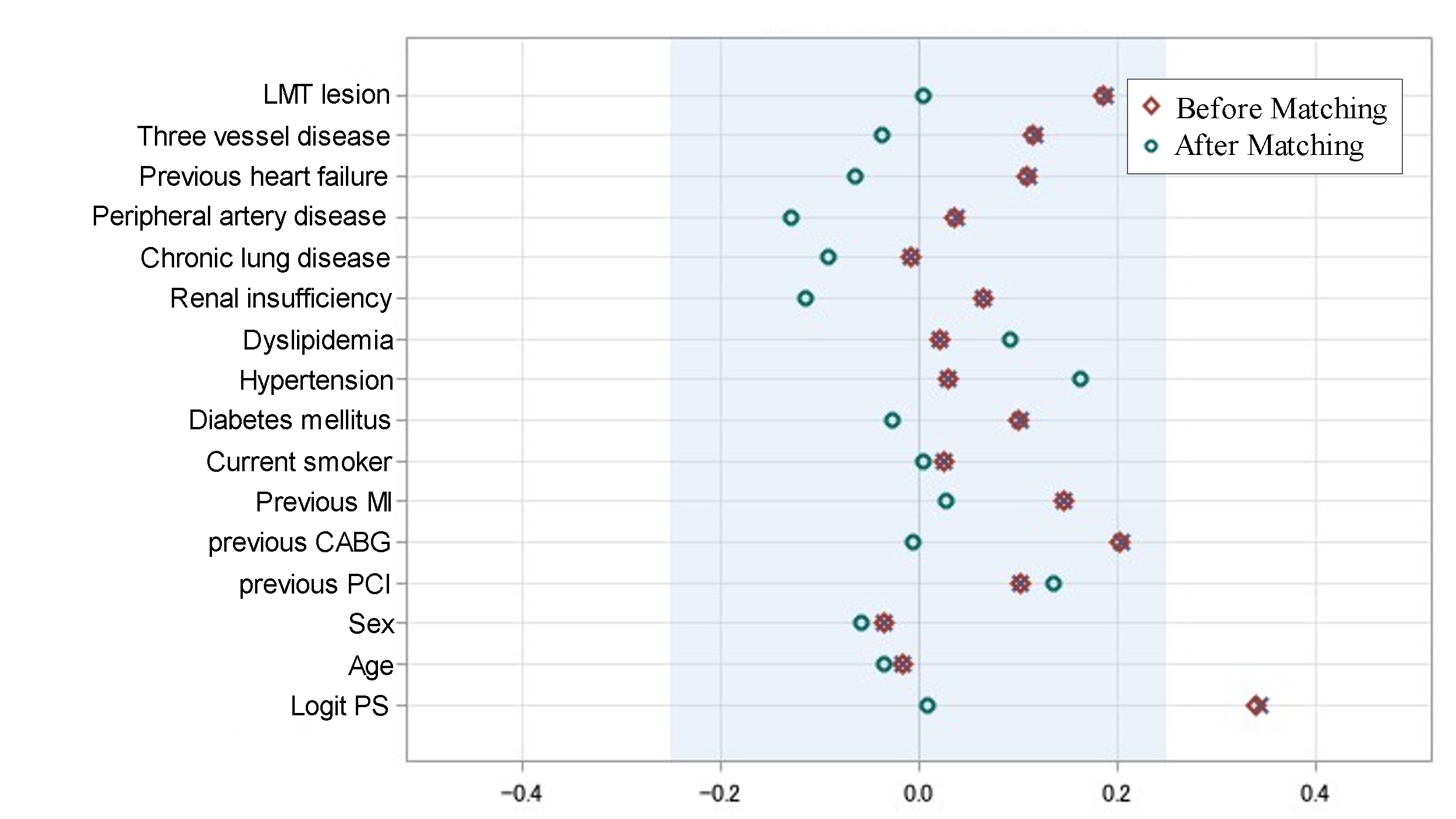


**Variables Included in the Propensity Score (PS):** age, sex, previous percutaneous coronary intervention (PCI), previous coronary artery bypass grafting (CABG), previous myocardial infarction (MI), diabetes mellitus, hypertension, dyslipidemia, current smoking, renal insufficiency, chronic lung disease, peripheral artery disease, previous heart failure, three-vessel disease, and left main trunk (LMT) lesion.
